# Path Analysis Results on Bone Markers That Influence Accelerated Bone Remodeling: An Experimental Study

**DOI:** 10.1002/cre2.70326

**Published:** 2026-04-22

**Authors:** Renie Kumala Dewi, Sri Oktawati, Asdar Gani, Eko Suhartono, Nurlindah Hamrun, Rasmidar Samad, Nurhayaty Natsir, Maharani Laillyza Apriasari, Hasanuddin Hasanuddin

**Affiliations:** ^1^ Department of Pediatric Dentistry, Faculty of Dentistry Lambung Mangkurat University Banjarmasin Indonesia; ^2^ Department of Periodontology, Faculty of Dentistry Hasanuddin University Makassar Indonesia; ^3^ Department of Medical Chemistry and Biochemistry, Faculty of Medicine Lambung Mangkurat University Banjarmasin Indonesia; ^4^ Department of Oral Biology, Faculty of Dentistry Hasanuddin University Makassar Indonesia; ^5^ Department of Dental Public Health and Preventive Dentistry, Faculty of Dentistry Hasanuddin University Makassar Indonesia; ^6^ Department of Conservative Dentistry, Faculty of Dentistry Hasanuddin University Makassar Indonesia; ^7^ Department of Oral Medicine, Faculty of Dentistry Lambung Mangkurat University Banjarmasin Indonesia; ^8^ Master of Dental Sciences Study Program, Faculty of Dentistry Hasanuddin University Makassar Indonesia

**Keywords:** Black Soldier Fly (*Hermetia illucens*), bone regeneration, demineralized dentin matrixs, nanochitosan, path analysis

## Abstract

**Objective:**

We examined the effectiveness of combining nanochitosan Black Soldier Fly (BSF) pupae and demineralized dentin matrix (DDM) for accelerated bone remodeling by path analysis of osteoblasts, OPG, osteocalcin (OCN), osteoclasts, and RANKL.

**Methods:**

Laboratory research using eighteen samples of male guinea pigs that met the inclusion and exclusion criteria, the lower left incisor teeth were extracted, then taken randomly and divided into a control group (C), which applied polyethylene glycol (PEG) gel as a placebo and sutured with nonabsorbable silk, and a treatment group (T) applied a combination of nanochitosan BSF pupae and DDM gel, then sutured with nonabsorbable silk. Samples were euthanized on days 7, 14, and 21 to examine osteoblasts, OPG, OCN, osteoclasts, and RANKL. Data were analyzed using One‐way ANOVA (*p* < 0.05). Pathway analysis was performed using a goodness‐of‐fit model and effect size, testing the hypothesis that OPG has a direct and dominant influence on RANKL and OCN, resulting in increased osteoblasts and decreased osteoclasts.

**Results:**

There was a decrease in the number of osteoclasts, RANKL expression, and an increase in the number of osteoblasts, OPG, and OCN expression between the C and T groups. One‐way ANOVA analysis showed a significant difference (0.000) (*p* < 0.05). The pathway analysis that contributed most significantly in reducing osteoclasts and increasing osteoblasts, thereby accelerating bone remodeling, was OPG affecting OCN (0.952), rather than OPG affecting the RANKL pathway (−0.001).

**Conclusions:**

The combination of nanochitosan BSF pupae and DDM has a maximum impact on bone remodeling, so that it can be used as an alternative bone graft material for socket healing treatment and accelerating alveolar bone regeneration.

## 1 Background

Tooth extraction is the most commonly performed treatment for dental caries, periodontal disease, trauma, and other conditions that potentially cause alveolar bone defects. Post‐extraction wound healing involves the recovery of hard tissue in the alveolar bone and soft tissue, particularly the gingival epithelium and connective tissue (Ozer and Inci 2024). The biochemical process of wound healing evolves in several phases (inflammatory response, cellular proliferation, synthesis of extracellular matrix components, and remodeling) that may overlap over time (Juodzbalys et al. 2019; Gonzalez et al. 2016). Clinically, post‐extraction bone loss can range from 5 to 7 mm, with approximately two‐thirds of the bone lost within the first 3 months. Excessive bone loss may impair the bone's self‐repair capacity, thus requiring supportive materials, such as bone grafts, to aid healing, prevent infection, and promote bone growth. A good bone graft must meet several criteria: it must be biocompatible (well tolerated by the body) and possess osteoconductive, osteoinductive, and osteogenic properties. Bone grafts can be stimulated by biomaterials containing osteoconductive and osteoinductive components. During bone healing, bone graft materials such as allografts, xenografts, and alloplasts can provide mechanical support to prevent bone defects, thereby accelerating the bone remodeling process (Bono et al. 2019; Compton and Lee 2014; Hamrun et al. 2025). Given various options for bone graft materials, this study proposes a gel formulation combining nanochitosan from Black Soldier Fly (BSF) pupae and demineralized dentin matrix (DDM) as a promising bone graft alternative to promote alveolar bone regeneration and socket healing by reducing osteoclasts and RANKL while enhancing osteoblasts, osteoblasts release osteoprotegerin (OPG), and osteocalcin (OCN), with OCN acting as a key regulatory factor.

One method for preserving bone dimensions is socket preservation, which can be achieved using bone graft materials (Bashir et al. 2022). Innovations in bone tissue engineering have led to the development of three‐dimensional scaffolds, such as biodegradable polymers, which can accelerate the replacement of damaged tissue or function as an extracellular matrix. Chitosan (CS) is one of the most widely used polymers. CS is a natural polysaccharide synthesized from chitin extracted from animal shells. The broad application of chitosan across various fields has encouraged the development of numerous studies utilizing chitosan, including physical modifications that involve reducing its size to nanoparticles. Nanoparticles offer advantages over similar materials due to their higher reactivity (Sam et al. 2022; Loo et al. 2022). However, chitosan exhibits certain limitations, including low mechanical strength, rigid and brittle membranes, and a lack of active sites that enhance membrane functionality. To overcome these limitations, chitosan can be modified by combining it with other natural materials, such as DDM, which is derived from human teeth. Bone and dentin have the same composition, consisting of 70% hydroxyapatite, 18% collagen, 10% body fluid, and 2% non‐collagen protein. Bone and dentin contain several growth factors that enable dentin to stimulate osteoinduction, osteoconduction, and blood vessel formation. DDM has been widely used in dental research because it is readily available in large quantities, of high quality, and at a low cost (Gao et al. 2019; Um et al. 2017).

The exoskeleton of the BSF (*Hermetia illucens*) is composed of approximately 35% chitin, which is notable during the pupal stage. Thus, chitin is a promising source of chitin. Both prepupal and pupal developmental phases are suitable for chitin extraction due to the presence of shed exuviae and pupal casings rich in this polysaccharide. Additionally, BSF prepupae exhibit high concentrations of essential amino acids, including lysine, leucine, and valine, which enhances their nutritional and industrial value. Functional amino acids play a crucial role in regulating bone remodeling, as they influence key metabolic pathways essential for maintaining bone homeostasis. Chitosan exhibits a high binding capacity, making it suitable for use as an absorbent and drug delivery medium, and is composed of amino‐NH2 groups and hydroxyl groups(–OH). Chitosan is a cationic polysaccharide composed of repeating D‐glucosamine‐glucosamine and N‐acetyl‐D‐glucosamine units. Its structural properties resemble those of hyaluronic acid, a glycosaminoglycan (GAG) present in the extracellular matrix. HA is a key macromolecule in tissue repair (Awad and Alzubaidi 2022; Zhang et al. 2017; Wang et al. 2023; Takeuchi et al. 2024; Iba and Wardhana 2024; Rajinikanth B et al. 2024).

A key innovation of this study is the utilization of a composite material consisting of NBSF and DDM to enhance the process of bone remodeling. Previous studies have explored the use of chitosan and nanochitosan; however, their application in combination with DDM has not been investigated. As reported by Zhang et al. (2017), the application of chitosan modulated bone remodeling by enhancing OPG expression and reducing RANKL levels. Gao et al. (2019) concluded that the application of DDM can accelerate bone regeneration, as dentin contains several growth factors that can stimulate osteoinduction, osteoconduction, and blood vessel formation (Gao et al. 2019; Zhang et al. 2017).

Another novelty is that several previous studies have used OPG/RANKL parameters to measure the increase in osteoblasts and decrease in osteoclasts, and bone remodeling. There are several other biomarkers, including OCN. This study employed a more comprehensive methodological approach by utilizing path analysis to statistically evaluate the direct and indirect effects between variables, specifically to establish causal relationships. We examined whether OPG can reduce RANKL and increase OCN, thereby enhancing osteoblast activity and reducing osteoclast activity. This approach helps determine statistically relevant components underlying the event under study, validates intricate theoretical frameworks, and assesses the degree of influence of the observed model (Fitria and Gunawan 2024; Yuza et al. 2014).

This study aimed to investigate the effects of combined nanochitosan BSF pupae and DDM on Socket preservation after tooth extraction by analyzing the biomolecular pathways involving osteoblasts, osteoclasts, OPG, RANKL, and OCN. The research questions to be answered in this study are as follows: (1) Does the application of a combination of nanochitosan BSF pupae and DDM increase the number of osteoblasts, OPG, and OCN while simultaneously decreasing the number of osteoclasts and RANKL expression during post‐extraction bone remodeling? (2). What is the causal relationship between OPG, RANKL, and OCN in the modulation of the balance of osteoblast and osteoclast activity during the remodeling process? This study provides a novel and innovative alternative for bone graft materials and offers an integrated understanding of the biomolecular mechanisms of accelerated bone regeneration. These findings are expected to contribute to the development of natural biomaterial‐based tissue engineering scaffolds, which are more effective and easily accessible for clinical applications in dentistry, particularly in socket preservation efforts, and to accelerate alveolar bone healing.

## 2 Material and Methods

### 2.1 Ethical Approval and Consent to Participate

All research procedures using guinea pig samples have been approved by the Joint Ethics Committee of the Faculty of Dentistry, Hasanuddin University, and the Dental and Oral Hospital of Hasanuddin University, Makassar, Indonesia (Approval Number 0108/PL.09/KEPK FKG‐RSGM UNHAS/2023). All methods were carried out in accordance with applicable guidelines and regulations.

### 2.2 Study Design

The research employs a post‐test‐only control group design with a sample of eighteen male guinea pigs (*Cavia cobaya*). The left mandibular incisor extraction was performed, and the subjects were divided into two groups: a control group (C), which received a polyethylene glycol (PEG) gel as a placebo and was sutured with non‐absorbable sutures; and a treatment group (T), which received a gel containing a combination of nanonchitosan BSF pupae, and DDM, followed by suturing with non‐absorbable sutures. The specimens were euthanized on days 7, 14, and 21 for histological analysis of osteoblasts, osteoclasts, and immunohistochemical (IHC) examination of OPG, OCN, and RANKL expression levels. The data were analyzed using One‐Way ANOVA (*p* < 0.05). Additionally, pathway analysis was conducted utilizing a goodness‐of‐fit model to assess direct and dominant effects, specifically examining how OPG influences RANKL and OCN levels. The results aim to determine whether osteoblast activity increases and osteoclast activity decreases following treatment, providing insights into bone regeneration processes in post‐extraction sites.

### 2.3 Sample Size

This study involved male guinea pigs (*Cavia cobaya*) that met specific inclusion and exclusion criteria. The sample size was calculated using a formula for unpaired comparative analysis with multiple measurements, resulting in a total of eighteen guinea pigs. The samples were randomly divided into two groups: the control group (C) (*n* = 9) and the treatment group (T) (*n* = 9). Random numbers were generated using the standard = RAND () function in Microsoft Excel.

### 2.4 Inclusion and Exclusion Criteria

The guinea pig samples were obtained from the Veterinary Teaching Hospital of Universitas Airlangga. The animals were included in the study if they were deemed healthy by a veterinarian. The animals were included in the study: the male guinea pigs at the time of the experiment were 2.5 to 3 months old, weighing 200–300 g, and were used. Animals were excluded if any guinea pigs died during the 7‐day acclimatization period before the experiment.

The guinea pig (*Cavia cobaya*) serves as a suitable experimental model because of its physiological and reproductive similarities to mammals, as well as its immunological and pathological responses that are comparable to those of humans. Mandibular incisors were selected for extraction because they are longer and larger than the maxillary teeth, making extraction easier. The guinea pig lacks both canine and premolar teeth, resulting in a diastema between the anterior and posterior teeth. The diastema between the anterior and posterior teeth, along with the more compact mandible, facilitates access to the anterior teeth (Sa'diyah et al. 2020).

### 2.5 Preparation of Nanochitosan and DDM Gel

BSF pupae are obtained from BSF farms in South Kalimantan, Indonesia, and then processed into chitosan. Chitosan derived from BSF pupae was produced in‐house under laboratory through sequential demineralization, deproteinization, and depigmentation processes to obtain chitin, followed by a deacetylation step to convert chitin into chitosan. The resulting chitosan achieved an 80% deacetylation rate, confirming its high purity. Chitosan was converted into nanochitosan using a magnetic stirrer to obtain spherical particles with a size of 204.9 nm. The DDM was prepared from human tooth roots extracted from alveolar sockets, blended (Tencan planetary ball mill, Japan) to obtain fine particles, and subjected to a demineralization process and freeze drying (KFD‐P F Series, Pilot Freeze Dryers, Korea) under vacuum conditions < 20 Pa and temperature (−80°C) until frozen and then dried for 18–24 h until the remaining water content was 5% and sterilized using gamma rays. The gel preparation was applied to the socket after tooth extraction by mixing nanochitosan BSF pupae powder and DDM (ratio 50:50) with PEG 400 and PEG 4000 until homogeneous.

### 2.6 Experimental Procedures

**Figure 1:**
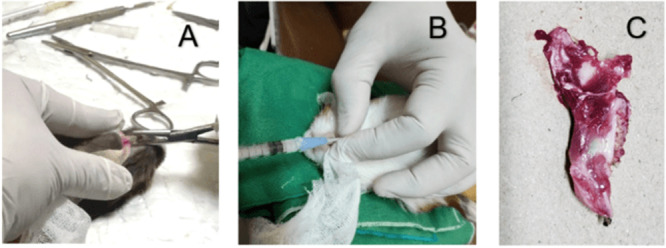
Sample guinea pig procedure (A). Tooth extraction with a needle holder (B). Application of material using a plastic syringe (C) Mandibular jaw dissection.

The guinea pigs used in the study will first be acclimated to the laboratory environment by maintaining the ideal cage temperature for guinea pigs, which is 20°C–26°C. During the study, the guinea pigs will be fed a standard diet consisting of vegetables supplemented with vitamin C and other vitamins, as well as 20–35 g of crude fiber per day. Eighteen male guinea pigs were intramuscularly injected with ketamine (Bernofarm, Indonesia) (50 mg/kg body weight) and xylazine (CXBT & ShowVet, Henan, China) (5 mg/kg body weight), followed by the extraction of the left mandibular incisor. In group C, the socket was filled with PEG gel using a plastic syringe and then sutured using non‐absorbable silk suture. In group T, BSF pupae nanochitosan and DDM were applied using a plastic syringe until the wound was filled, followed by suturing with non‐absorbable silk suture. Each group was euthanized on the 7th, 14th, and 21st days after the study. The euthanasia process was initiated with an intraperitoneal injection of ketamine at a lethal dose (4 times the anesthetic dose or 0.4 mL/400 grams of body weight), administered in the lower abdominal region until the guinea pig was deceased. Guinea pigs were considered alive when cardiac activity was still detectable, and a blink reflex was elicited upon tactile stimulation of the ocular surface. Following euthanasia, tissue samples were collected by cutting the mandible in the extraction socket region, vertically along the mesial and distal sides (Hakim 2022). Alveolar bone was taken from the socket area after tooth extraction to prepare paraffin blocks and slide specimens, which were then used to assess the bone regeneration process (Figure 1). Following euthanasia, the guinea pigs were subjected to burial. The animals’ bodies were first cleaned thoroughly before being buried at a depth of 25–75 cm (Hakim 2022).

### 2.7 Histological Analysis

The following parameters were assessed: the number of osteoblasts and osteoclasts, expression of RANKL, OPG, and OCN. Histopathological examination was performed using Hematoxylin and Eosin (HE) staining to observe the presence of osteoblasts and osteoclasts and to quantify their numbers. This was followed by IHC staining to assess the number of osteoblasts expressing RANKL, OPG, and OCN. IHC staining was performed on bone tissue sections using monoclonal antibodies against RANKL, OPG, and OCN. Quantification of osteoblasts, osteoclasts, and the expression levels of RANKL, OPG, and OCN was carried out using a light microscope (Olympus, Japan) at 400× magnification in the apical third region of the tooth socket, with counts taken from five fields of view by researchers, analysts, and clinical pathology specialists.

### 2.8 Statistical Analysis and Path Analysis

All measurements of osteoblasts, osteoclasts, OPG, RANKL, and OCN were tabulated and analyzed using one‐way ANOVA with a significance level set at *p* < 0.05. Additionally, model fit, effect size, and hypothesis testing were conducted to evaluate direct effects and dominant pathways, particularly examining how OPG influences RANKL and OCN through pathway analysis. This approach aims to determine the effects of increased osteoblast activity and decreased osteoclast activity. Pathway analysis enables the assessment of both direct and indirect effects among variables and helps identify causal relationships between them.

## 3 Result

### 3.1 Comparison Between Treatment and Control Groups

In this study, data were collected by examining the number of osteoblasts and osteoclasts as well as the expression levels of OPG, OCN, and RANKL on days 7, 14, and 21. The mean and standard deviation values of osteoblasts, OPG, and OCN were higher in the T group than in the C group, whereas RANKL and osteoclast values were lower in the T group than in the C group.

The decrease in the number of osteoclasts in the T group on day 21 (1.000 ± 0.0000) was the lowest among all groups, while the increase in the number of osteoblasts in the T group on day 21 (13.000 ± 2.0000) was the highest compared to the other groups (Table 1). Based on One‐Way ANOVA, the number of osteoblasts, expression levels of OPG and OCN, number of osteoclasts, and RANKL expression on days 7, 14, and 21 were significantly different (*p* < 0.05), with a *p*‐value of 0.000 across all groups.

**Table 1:** Mean ± standard deviation and significance of osteoblast, osteoclast, OPG, RANKL, and OCN.

	Group	Day 7	Day 14	Day 21	*p* value
	C	1.667 ± 0.5774	2.000 ± 1.0000	2.667 ± 0.5774	0.000*
Osteoblast	T	9.667 ± 11.547	12.000 ± 1.0000	13.000 ± 2.0000	
	C	8.000 ± 1.0000	7.667 ± 0.5774	7.000 ± 1.0000	0.000*
Osteoclast	T	3.333 ± 0.5774	1.667 ± 0.5774	1.000 ± 0.0000	
	C	1.333 ± 0.5774	2.667 ± 0.5774	7.300 ± 3.0000	0.000*
OPG	T	8.333 ± 0.5774	10.333 ± 0.5774	11.333 ± 1.000	
	C	7.667 ± 0.5774	6.667 ± 1.1547	3.500 ± 1.6523	0.000*
RANKL	T	3.667 ± 0.5774	1.667 ± 0.5774	1.000 ± 1.0000	
	C	1.667 ± 0.5774	3.000 ± 1.0000	7.500 ± 2.8513	0.000*
OCN	T	8.667 ± 0.5774	10.333 ± 0.5774	11.333 ± 2.0000	

* Significant = alpha = 5%.

Based on the results of the normality test using the Shapiro–Wilk test with a significance level of α = 0.05, all variables on each measurement day had significance values greater than 0.05 (*p* = 0.750), indicating a normal distribution. Homogeneity testing using Levene's test also showed significant *p*‐values greater than 0.05 (*p* = 1.791), indicating that the data had heterogeneous variance across all measurements. Therefore, the data were considered to be normally distributed, and subsequent statistical analyses were performed using a parametric One‐Way ANOVA test. The One‐Way ANOVA results revealed statistically significant differences (*p* < 0.05) in the number of osteoblasts, expression of OPG and OCN, number of osteoclasts, and expression of RANKL on days 7, 14, and 21 across all groups, with a *p*‐value of 0.000.

### 3.2 Histological and Immunohistochemical Findings

**Figure 2:**
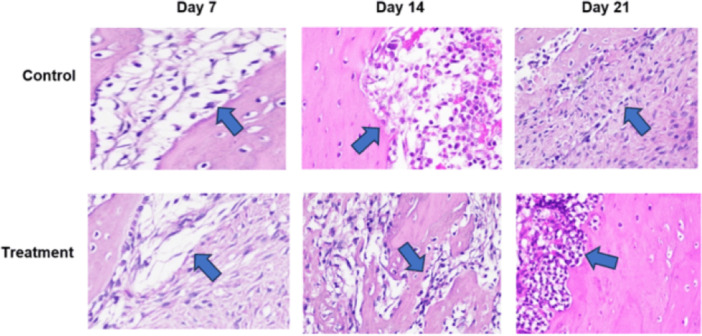
Histological observations of osteoblast cells in the C and T groups on days 7, 14, and 21 in alveolar bone specimens from the socket area following tooth extraction were observed at 400× magnification across five fields of view.

**Figure 3:**
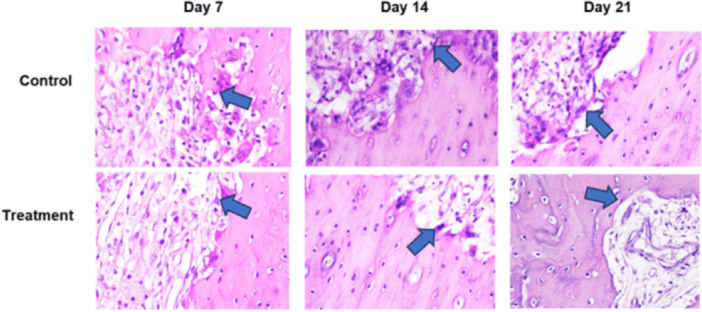
Histological observations of osteoclast cells in the C and T groups on days 7, 14, and 21 in alveolar bone specimens from the socket area following tooth extraction were observed at 400× magnification across five fields of view.

**Figure 4:**
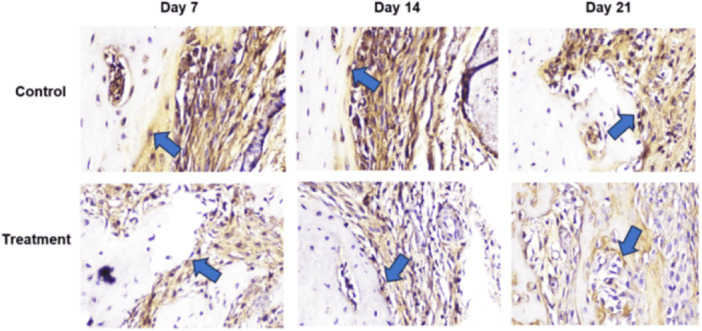
Histological observations of RANKL expression in the C and T groups on days 7, 14, and 21 in alveolar bone specimens from the socket area following tooth extraction, observed at 400× magnification across five fields of view.

**Figure 5:**
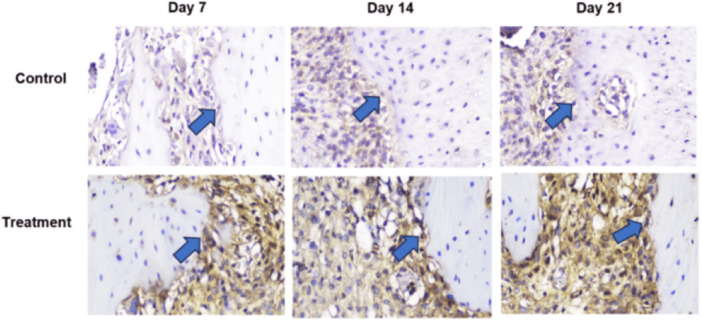
Histological observations of OPG expression in the C and T groups on days 7, 14, and 21 in alveolar bone specimens from the socket area following tooth extraction, observed at 400× magnification across five fields of view.

**Figure 6:**
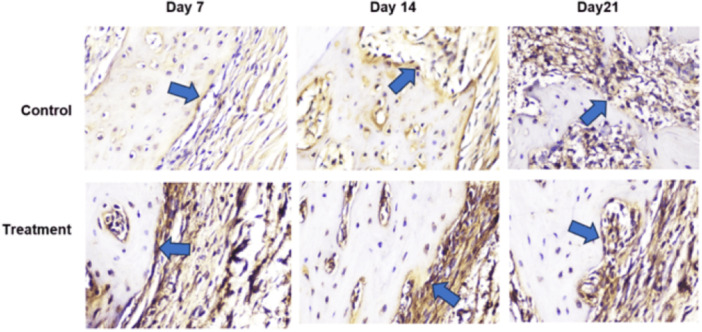
Histological observations of OCN expression in the C and T groups on days 7, 14, and 21 in alveolar bone specimens from the socket area following tooth extraction, observed at 400× magnification across five fields of view.

Based on histological examination using Hematoxylin and Eosin (HE) staining, observed sequentially from left to right in a zigzag pattern starting from the bottom upward, Figure 2 shows that the number of osteoblasts was higher in the T group than in the C group. Following tooth extraction, osteoblasts appeared as cuboidal cells with single, purple‐stained nuclei located on the surface of the bone matrix surrounding the socket. HE staining showed a greater number of osteoclasts in the C group than in the T group (Figure 3). Following tooth extraction, osteoclasts appear as large, motile cells with multiple reddish‐purple nuclei located around the socket. Figure 4 displays RANKL expression in osteoblasts, as revealed by IHC staining using a monoclonal anti‐RANKL antibody. Brown‐colored cells were more abundant around the post‐extraction socket in the C group than in the T group. As shown in Figure 5, OPG expression by osteoblasts, detected through IHC staining with a monoclonal anti‐OPG antibody, showed a higher number of brown‐colored cells in the C group than in the T group around the socket. As shown in Figure 6, OCN expression by osteoblasts, examined by IHC staining with a monoclonal anti‐OCN antibody, showed a greater number of brown‐colored cells in the T group than in the C group surrounding the post‐extraction socket.

### 3.3 Analysis of the Mediator Pathways in Bone Remodeling

**Figure 7:**
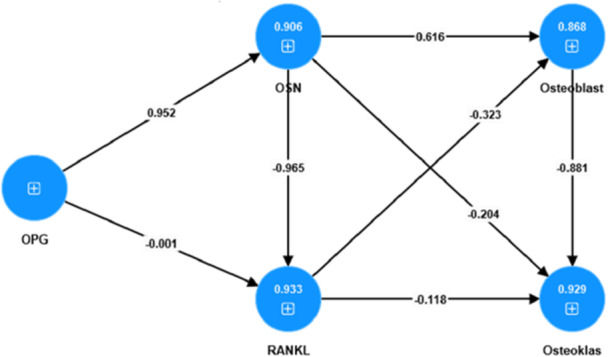
Structure of the path analysis model.

Path analysis was used to analyze the direct and indirect effects of increasing osteoblast number. Path analysis was performed using AMOS 24. The developed model must meet the goodness‐of‐fit criteria, which are achieved by entering the model and the data used in AMOS (Figure 7).

The path analysis model used in this study is as follows:

The Goodness of Fit Model was used to determine the extent to which the dependent variables (OPG, osteoblasts, and osteoclasts) could explain the variance in the independent variables (OCN, RANKL), or in other words, to assess the contribution of the dependent variables to the independent variables. In the partial least squares analysis, the Goodness of Fit Model is evaluated using the coefficient of determination (R‐Square) and the Q‐Square predictive relevance (Q^2^).

Based on Table 2, the contribution of OPG to OCN was 90.6% (0.906), whereas the remaining 9.4% was attributed to other variables not discussed in this study. The combined contribution of OPG and OCN to RANKL was 93.3% (0.933), with the remaining 6.7% being attributed to other variables outside the scope of this research. The contribution of OCN, RANKL, and osteoblasts to osteoclasts was 92.9% (0.929), while the remaining 7.1% was accounted for by other variables that were not examined in this study. Overall, the combined contribution of OPG, OCN, RANKL, and osteoblasts to osteoclasts (Q^2^) was 99.99% (0.999), with only 0.01% attributed to the other unexamined variables.

**Table 2:** Results of the goodness of fit model.

Goodness of fit model	R‐square
OCN	0.906
RANKL	0.933
Osteoblast	0.868
Osteoklas	0.929
Q^2^ Osteoklas	0.999

The influence of OPG, osteoclasts, and osteoblasts on RANKL and OCN was determined through effect size analysis, where an effect size mean value of 0.02–0.15 indicates a small effect, 0.15–0.35 indicates a moderate effect, and > 0.35 indicates a large effect. Direct effects hypothesis testing was used to examine whether OPG, osteoclasts, and osteoblasts had a direct influence on RANKL and OCN. The testing criterion states that, if the *p*‐value is ≤ the level of significance (alpha = 5%), the effect is considered statistically significant.

As shown in Table 3, the largest effect sizes were observed for the influence of OPG on OCN (9.612) and that of OCN on osteoblasts (0.616). Path coefficient hypothesis testing also demonstrated a strong effect of OPG on OCN (0.952) and OCN on osteoblasts (6.216). These findings suggest that higher levels of OPG result in an increase in OCN and, in turn, higher levels of OCN lead to an increase in osteoblasts. Conversely, lower levels of OPG result in a decrease in OCN, which subsequently leads to a reduction in osteoblast number. The significant effect of OPG on OCN yielded a *p*‐value of 0.000 (*p* < 0.05) and the impact of OCN on osteoblasts yielded a *p*‐value of 0.018 (*p* < 0.05). These results indicate that OPG has a statistically significant influence on OCN, as well as a significant effect on osteoblasts.

**Table 3:** Results of the effect size and path coefficient of OPG, osteoblasts, osteoclasts, RANKL, and OCN.

Influence	Between variables	Effect size	Path coefficient	*p* values
OPG	OCN	9.612	0.952	0.000*
OPG	RANKL	5.446	−0.001	0.997
OCN	RANKL	1.304	−0.965	0.000*
OCN	Osteoblast	6.216	0.616	0.018*
OCN	Osteoklas	0.033	−0.204	−0.456*
RANKL	Osteoblast	0.053	−0.323	0.210
RANKL	Osteoklas	3.741	−0.118	0.631
Osteoblast	Osteoklas	1.431	−0.888	0.000*

* Significant = alpha = 5%.

A significant effect was also observed in the influence of OCN on RANKL, with a *p*‐value of 0.000 (*p* < 0.05), as well as in the effect of OCN on osteoclasts, with a *p*‐value of −0.456 (*p* < 0.05), and the effect of osteoblasts on osteoclasts, with a *p*‐value of 0.000 (*p* < 0.05). These results indicated a statistically significant relationship between OCN and RANKL, OCN and osteoclasts, and osteoblasts and osteoclasts. A significant effect was observed in the influence of OCN on osteoclasts, as well as in the influence of osteoblasts on osteoclasts, as well as in the influence of OCN on osteoclasts, as well as in the influence of osteoblasts on osteoclasts. The results of path coefficient hypothesis testing showed negative values for the influence of OCN on RANKL (−0.965), OCN on osteoclasts (−0.204), and osteoblasts on osteoclasts (−0.888). These negative relationships indicated that higher levels of OCN reduced RANKL expression, whereas lower levels of OCN increased RANKL expression. Similarly, higher OCN levels led to a decrease in osteoclast numbers, whereas lower OCN levels resulted in an increase. Additionally, higher levels of osteoblasts are associated with a reduction in osteoclasts, whereas lower levels of osteoblasts correspond to an increase in osteoclast numbers.

**Figure 8:**
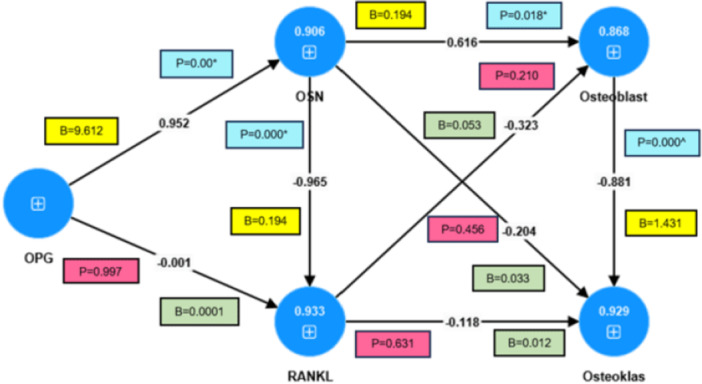
Path analysis results with path coefficient values.

Negative path coefficient values were also observed for the relationships between OPG and RANKL (−0.0001), RANKL and osteoblasts (−0.323), RANKL and osteoclasts (−0.118), and between osteoblasts and osteoclasts (−0.888). These results indicate that higher levels of OPG reduced RANKL expression, whereas higher levels of RANKL decreased both osteoblast and osteoclast activity. Additionally, higher levels of osteoblasts lead to a reduction in osteoclasts. Conversely, lower levels of OPG increase RANKL expression, lower levels of RANKL lead to increased osteoblast and osteoclast activity, and lower levels of osteoblasts increase osteoclasts. The results showed that the influence of OPG on RANKL had a *p*‐value of 0.997 (*p* < 0.05), the influence of RANKL on osteoblasts had a *p*‐value of 0.210 (*p* < 0.05), and the influence of RANKL on osteoclasts had a *p*‐value of 0.631 (*p* < 0.05). These findings indicated that the effects of OPG on RANKL, RANKL on osteoblasts, and RANKL on osteoclasts were not statistically significant. Based on the path coefficients presented in Tables 1 and 2, the overall causal relationships among the variables are illustrated in a path diagram. The constructed diagram shows the values associated with each variable. The pathway mechanisms of the variables are shown in Figure 8.

The dominant influence of osteoblasts, OPG, and osteoclasts on RANKL and OCN can be identified through the total coefficients with the highest absolute values, regardless of whether the coefficients are positive or negative. The values are presented in Table 4.

**Table 4:** Dominant influence of osteoblasts, OPG, and osteoclasts on RANKL, OCN.

Influence	Between variables	Effect size
OPG	OCN	0.952
OPG	RANKL	−0.919
OPG	Osteoblast	0.883
OPG	Osteoklas	−0.864
OCN	RANKL	−0.965
OCN	Osteoblast	0.928
OCN	Osteoklas	−0.908
RANKL	Osteoblast	−0.323
RANKL	Osteoklas	0.167
Osteoblast	Osteoklas	−0.881

The results indicated that the strongest total coefficient affecting RANKL was OCN, with a total coefficient of −0.965, indicating that OCN has the most dominant influence on RANKL. Similarly, OSN had the highest total coefficient (0.928) for osteoblasts, confirming its predominant effect on osteoblast activity. Furthermore, OSN demonstrated the most significant total coefficient (−0.908) for osteoclasts, establishing its dominant regulatory role in osteoclast modulation.

## 4 Discussion

The results of this study showed that the T group had the highest mean values for osteoblast number, OPG expression, and OCN levels, while showing the lowest mean values for osteoclast number and RANKL expression compared with the C group on days 7, 14, and 21. These findings support the hypothesis that osteoblast proliferation, OPG expression, and OCN levels are increased. In contrast, osteoclast number and RANKL expression decreased during the bone formation process during the remodeling phase. Furthermore, the study revealed a significant relationship between osteoblasts, osteoclasts, RANKL, OPG, and OCN following tooth extraction after administration of a combined treatment of BSF (*H. illucens*) nanochitosan and DDM on days 7, 14, and 21. Previous studies have shown that chitosan exhibits hemostatic, antibacterial, and anti‐inflammatory activities (Awad and Alzubaidi 2022).

Chemically, chitosan is a linear polysaccharide composed of β‐(1,4)‐2‐amino‐2‐deoxy‐D‐glucopyranose chains, with a structure similar to that of GAGs, which play a critical role in wound healing. When applied to wound sites, the cationic properties of chitosan interact with the anionic characteristics of the cell surface, creating electrostatic adhesion. This interaction facilitates the binding of negatively charged red blood cells and platelets to the positively charged chitosan molecules at the wound surface. This interaction accelerates the hemostatic process and initiates the inflammatory phase, which lasts until approximately day 7. During this phase, chitosan exerts an antibacterial effect, leading to bacterial cell death (Iba and Wardhana 2024; Rajinikanth B et al. 2024). Chitosan used in this study exhibited a high degree of deacetylation (80%). Consistent with Hakim's (2022) findings, chitosan with a higher degree of deacetylation exhibited superior antibacterial efficacy compared with chitosan with a lower degree of deacetylation. An elevated degree of deacetylation correlates with an increased availability of active chitosan molecules that inhibit bacterial growth (Iba and Wardhana 2024; Rajinikanth B et al. 2024; Umar and Sujud 2020).

**Figure 9:**
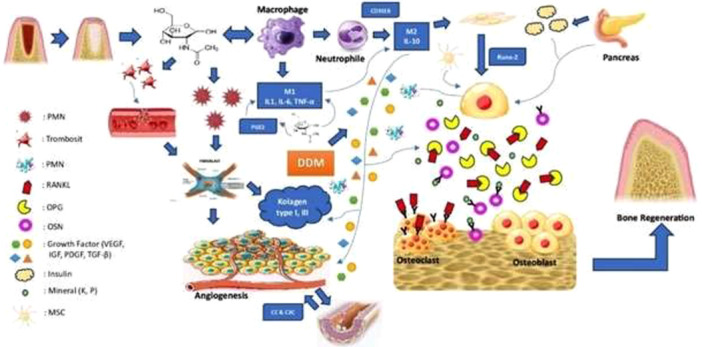
Pathway mechanism of bone regeneration with the combination of nanochitosan BSF pupae and DDM. Nanochitosan, upon contact with inflamed cells or tissues, secretes N‐acetylglucosamine (NAG) and binds to macrophages, leading to increased fibroblast proliferation and accelerated angiogenesis. DDM stimulates osteoblasts through the action of growth factors, promoting the expression of OPG and thereby preventing the binding of RANKL to RANK, which enhances osteoblast activity. In a parallel pathway, the pancreas secretes insulin, which stimulates osteoblast formation by inducing OCN expression.

Macrophages appear 24 h after pro‐inflammatory activity with a polarity of M1 phenotype (CD86+) that releases anti‐inflammatory cytokines such as IL‐1β, IL‐6, and TNF‐α, induced by nitric oxide synthase (iNOS), and are involved in pathogen elimination, inflammatory cytokine release, and release of a Th1 type reaction. Macrophages appear 24 h after pro‐inflammatory activity with M1 phenotype polarity (CD86+) that release anti‐inflammatory cytokines such as IL‐1β, IL‐6, and TNF‐α, which are induced by iNOS, and are involved in pathogen elimination, inflammatory cytokine release, and Th1 type reaction release. Increasing the number of macrophages in the inflammatory phase is also assisted by osteopontin (OPN). OPN works on the organism by playing a key role in the secretion levels of interleukin‐10 (IL‐10), interleukin‐12 (IL‐12), interleukin‐3 (IL‐3), interferon‐γ (IFN‐γ), integrin αvB3, nuclear factor kappa B (NF‐kB), macrophages, and T cells, regulating osteoclast function and affecting the CD44 receptor. The inflammatory phase begins within 0–7 days following injury, during which acute inflammatory cells and neutrophils eliminate debris and bacteria through a process known as phagocytosis. Subsequently, neutrophils undergo phagocytosis by the macrophages. During this phase, macrophages release cytokines and growth factors, as they assume the predominant role of polymorphonuclear cells in phagocytosing debris and necrotic tissues. These include proliferative phase‐initiating Transforming Growth Factor‐Beta (TGF‐β), Transforming Growth Factor‐Alpha (TGF‐α), Fibroblast Growth Factor (FGF), and Vascular Endothelial Growth Factor (VEGF) (Figure 9) (Umar and Sujud 2020; Dewi et al. 2024; Gani 2022; Gomes et al. 2019). The cytokines in this phase are categorized as anti‐inflammatory cytokines (M2), such as IL‐4, IL‐10, and IL‐13, and pro‐inflammatory cytokines (M1), including TNF‐α, IL‐1, IL‐6, IL‐8, and IL‐γ. According to Gani (2022), applying chitosan on days 7, 14, and 21 dramatically decreased the production of the pro‐inflammatory cytokine IL‐1, limiting excessive inflammation while also upregulating BMP‐2 to promote osteogenesis and osteoinduction. It is essential to note that pro‐inflammatory cytokines produced during the inflammatory phase can induce osteoblasts to increase RANKL production, while simultaneously suppressing OPG expression. OPG, which inhibits the binding of RANKL to RANK, thereby increasing osteoblast activity and decreasing osteoclast activity. The bone remodeling process is initiated by this change in cellular equilibrium, marked by an increase in osteoblast number and a decrease in osteoclast differentiation (Umar and Sujud 2020; Gani 2022; Pudji and Retno 2019; Wang et al. 2023).

In this study, the progressive daily increase in osteoblast numbers and OPG expression caused the observed decrease in osteoclast numbers and RANKL expression. The path coefficient results corroborate this assertion, as they suggest that OPG is negatively correlated with RANKL. Specifically, elevated levels of OPG result in decreased RANKL expression. Additionally, the path coefficient for the effect of RANKL on osteoblasts also exhibited a negative relationship, suggesting that osteoblast proliferation was facilitated by reduced RANKL levels (Table 3).

The decreased RANKL expression observed in the treatment group at days 7, 14, and 21 (Table 1) indicated that the combination of nanochitosan BSF pupae and DDM inhibited osteoclast formation and activation. RANKL plays a key role in osteoclast differentiation and activation. Chitosan applied to the post‐extraction socket can stimulate upregulation of TGF‐β1, PDGF, FGF2, and BMP mRNA expression. These growth factors stimulate the proliferation of fibroblasts. The upregulation of TGF‐β1 contributes to the enhanced stimulation of osteoblast activity. The late inflammatory phase is characterized by the release of growth factors, including TGF‐β, TGF‐α, FGF, and VEGF. In addition to the function of nanochitosan BSF pupae during the inflammatory phase, DDM contributes to the release of osteoinductive growth factors that induce bone formation. The organic matrix of DDM is composed of a variety of growth factors, including IGF, FGF, TGF‐β1, VEGF, and BMP. The proliferative phase is initiated and accelerated by the secretion of these factors (Figure 9) (Erlyn et al. 2024; Kazimierczak et al. 2021).

This study is consistent with an in vitro study conducted by Kazimierczak et al. (2021), which demonstrated that after 7 days, macrophages cultured on the surface of chitosan released significantly higher levels of IL‐4 and TGF‐β1 than M0, M1, and M2 macrophages. IL‐4 is an osteotropic factor that plays a crucial role in bone metabolism. Moreover, IL‐4 reduced the levels of osteoclast precursors. In addition, elevated levels of TGF‐β1 enhance the expression and secretion of OPG, a decoy receptor for RANKL, thereby suppressing RANKL–RANK‐mediated osteoclastogenesis (Both et al. 2025; Ding et al. 2020).

This study is consistent with the results of Ding et al. (2020), who reported that the application of DDM can increase the alveolar bone height ratio and promote new bone formation in locations where dental implants are inserted by the fourth week. Bone and dentin are mineralized tissues with nearly identical chemical composition. They are composed of an estimated 18% collagen, 2% non‐collagenous proteins (NCPs), 70% hydroxyapatite (HA), and 10% body fluids. The matrix serves as a reservoir for growth factors, including Bone Morphogenetic Protein (BMP), Transforming Growth Factor‐β (TGF‐β), Insulin‐Like Growth Factor (IGF), and Basic Fibroblast Growth Factor (bFGF). Several NCPs, such as OCN and OPN, are commonly found in both bone and dentin. BMPs, FGFs, type I collagen, and type III collagen are extracellular matrix components that are classified as NCPs in the bone and dentin matrices. Dentin can actively promote osteoinduction, osteoconduction, and vascularization processes due to the presence of specific growth factors (Koga et al. 2016; Um et al. 2017; Nagy and Penninger 2015).

Although the bone remodeling pathway is largely influenced by the OPG‐RANKL and OCN bonds, OPN also plays a crucial role in bone metabolism and homeostasis. OPN is involved in biological activity through its participation in the proliferation, migration, differentiation, and adhesion of several bone‐related cells, including chondrocytes, synoviocytes, osteoclasts, osteoblasts, and bone marrow mesenchymal stem cells (MSCs). OPN is produced by osteoblasts and osteocytes to bind osteoclasts to the bone surface. The interaction between OPN and integrin α3 on osteoclasts is crucial for forming a sealing zone that allows the cells to release acids and enzymes to break down old bone tissue (Icer and Gezmen‐Karadag 2018).

Path analysis results indicate that the OPG pathway has the strongest influence on OCN. The path coefficient indicated a positive (direct) relationship between OPG and OCN, suggesting that an increase in OPG is associated with a corresponding increase in OCN. OCN, an NCP in the bone matrix, is a critical indicator of bone formation. It is also referred to as bone GLA (gamma‐carboxyglutamic acid) protein (BGP). Osteoblasts synthesize collagen and are secreted into the interstitial fluid of the primary supporting bone tissue. OCN (osteocalcin) is a protein produced by osteoblasts that is dependent on vitamins K and D. It is one of the three proteins synthesized by osteoblasts that binds to hydroxyapatite in the bone matrix. RANK is expressed in hematopoietic osteoclast progenitor cells. OPG and RANK are receptors that exhibit similar affinities for RANKL. OPG produced by osteoblasts functions as a decoy receptor for RANKL, preventing it from binding to and activating RANK. This finding is consistent with the results of the present study, which revealed that the path analysis identified OCN as the variable with the highest total coefficient influencing RANKL. Therefore, OCN was the dominant variable affecting RANKL expression. Similarly, OCN was responsible for the highest total coefficient influencing osteoblasts. Therefore, OCN had the most influential effect on osteoblasts, and the highest total coefficient for the osteoclast variable was attributed to OCN, suggesting that OCN has the most influential effect on osteoclasts. The treatment group that received a combination of nanochitosan BSF pupae and DDM exhibited a decrease in osteoclast count and RANKL expression, as well as an increase in osteoblast count and the expression of OPG and OCN, as indicated in Sections Results, 3 and Conclusions, 4 above (Table 1) (Prahasanti et al. 2019; Dewi et al. 2025; Kanazawa 2015; Oktawati et al. 2018).

OCN (osteocalcin) is recognized as a gamma‐carboxyglutamic acid (Gla) protein, which is the most abundant NCP in bone tissue and is synthesized by osteoblasts during bone formation. OCN is a key factor in bone endocrinology. Osteoblasts possess functional insulin receptors, and insulin treatment has been shown to stimulates osteoblast proliferation and differentiation. In an alternative signaling pathway, the pancreas secretes insulin, which promotes OCN expression and osteoblast differentiation by inhibiting Runx2, resulting in the accumulation of carboxylated OCN in the bone matrix. Insulin activates osteoclasts and accelerates bone turnover by increasing OPG/RANKL ratio. OCN also has an endocrine function, regulates systemic glucose homeostasis, and plays a crucial role in the interaction between the bone, pancreas, and adipose tissue. Although several clinical studies have suggested that OCN may be involved in systemic glucose homeostasis, there is currently no direct evidence that OCN regulates glucose metabolism in humans (Kanazawa 2015; Oktawati et al. 2018; Ghuchani et al. 2024).

This finding is also consistent with that of the present study, in which path analysis revealed that the variable with the highest total coefficient influencing RANKL was OCN. OCN was the most significant variable influencing RANKL and also had the highest total coefficient influencing osteoblasts. As a result, OCN had a considerable impact on osteoblasts and the highest total coefficient influencing osteoclasts, suggesting that OCN is the most important factor influencing osteoclasts. As a result, administering nanochitosan BSF pupae and DDM together can increase the expression of OPG and OCN and the osteoblast count, while decreasing the expression of RANKL and osteoclast total.

Complications following tooth extraction include pain, discomfort, bleeding, insensitivity to touch, or loss of function. Applying this material is expected to reduce the incidence of post‐tooth extraction complications, according to research. However, this study has limitations. Considering the limitations of this study, it is reasonable to conclude that there was a greater increase in the OPG/RANKL ratio in the T group than in the C group, indicating accelerated bone remodeling following the application of the nanochitosan BSF pupae and DDM combination. However, further research is needed to explore the acceleration of bone remodeling by examining other bone formation mediators, such as OPN. OPN is an important extracellular matrix protein in bone remodeling that acts as an attachment bridge between osteoclast cells and the mineral matrix. Further studies with a longer observation period, such as 28–30 days, are needed, as the bone remodeling phase can last up to a year.

## 5 Conclusion

In conclusion, we have found that the application of a gel combination nanochitosan of BSF (*H. illucens*) pupae and DDM significantly reduced the number of osteoclasts and RANKL expression, while increasing the number of osteoblasts and the expression of OPG and OCN. The dominant pathway for enhancing osteoblast activity and reducing osteoclast numbers through RANKL suppression was mediated by OCN, suggesting that this combination may serve as a potential alternative bone graft material for socket healing and acceleration of alveolar bone regeneration.

## Author Contributions

Conception and design: Renie Kumala Dewi, Sri Oktawati, Asdar Gani, and Eko Suhartono. Data acquisition and authenticity of raw data: Nurlindah Hamrun and Nurhayati Natsir. Data analysis: Rasmidar Samad and H. Hasanuddin. Data interpretation: Maharani Laillyza Apriasari. Final manuscript approval: All authors.

## Funding

The authors have nothing to report.

## Ethics Statement

This study was approved by the Joint Ethical Committee of the Faculty of Dentistry of Hasanuddin University and the Dental Hospital Teaching of Hasanuddin University, Makassar, Indonesia (Approval No. 0108/PL.09/KEPK FKG‐RSGM UNHAS/2023).

## Consent

The authors have nothing to report.

## Conflicts of Interest

The authors declare no conflicts of interest.

## Data Availability

The data presented in this study are available upon request from the corresponding authors.
